# The Impact of Pre-operative Nutritional Status on Outcomes Following Congenital Heart Surgery

**DOI:** 10.3389/fped.2019.00429

**Published:** 2019-10-23

**Authors:** Carey Yun Shan Lim, Joel Kian Boon Lim, Rajesh Babu Moorakonda, Chengsi Ong, Yee Hui Mok, John Carson Allen, Judith Ju-Ming Wong, Teng Hong Tan, Jan Hau Lee

**Affiliations:** ^1^Department of Pediatrics, National University Hospital, Singapore, Singapore; ^2^Children's Intensive Care Unit, Department of Pediatric Subspecialties, KK Women's and Children's Hospital, Singapore, Singapore; ^3^Department of Biostatistics, Singapore Clinical Research Institute, Singapore, Singapore; ^4^Nutrition and Dietetics, KK Women's and Children's Hospital, Singapore, Singapore; ^5^Centre for Quantitative Medicine, Duke-NUS Medical School, Singapore, Singapore; ^6^Cardiology Service, Department of Pediatric Subspecialties, KK Women's and Children's Hospital, Singapore, Singapore

**Keywords:** nutrition, congenital heart disease, congenital heart surgery, weight-for-age z-score, height-for-age z-score, body mass index-for-age z-score, outcomes

## Abstract

**Aims and Objectives:** Malnutrition is common in children with congenital heart disease and may contribute to adverse outcomes. This study evaluates the impact of pre-operative nutritional status on outcomes after congenital heart surgery.

**Methods:** We conducted a retrospective cohort study enrolling children under 10 years old who underwent congenital heart surgery at a tertiary children's hospital from 2012 to 2016. Patients who had patent ductus arteriosus ligation only, genetic syndromes, or global developmental delay were excluded. Outcome measures included 30-day mortality, intensive care unit (ICU) length of stay (LOS), hospital LOS, duration of mechanical ventilation, and number of inotropes used post-operatively. We performed univariate/multivariable logistic regression analysis, adjusting for age, cyanotic cardiac lesion, co-morbidity, and Risk Adjustment for Congenital Heart Surgery (RACHS-1) score.

**Results:** Three hundred two children of median age 16.2 [interquartile range (IQR) 3.1, 51.4)] months were included. The most common cardiac lesions were ventricular septal defect (27.8%), atrial septal defect (17.9%), and Tetralogy of Fallot (16.6%). Median weight-for-age z-score (WAZ) was −1.46 (IQR −2.29, −0.61), height-for-age z-score (HAZ) was −0.94 (IQR −2.10, −0.10), and body mass index (BMI)-for-age z-score (BAZ) was −1.11 (IQR −2.19, −0.30). In multivariable analysis, there was an increased risk of 30-day mortality for WAZ ≤−2 vs. WAZ >−2 [adjusted odds ratio (aOR): 4.01, 95% CI: 1.22, 13.13; *p* = 0.022]. For HAZ ≤-2 vs. HAZ > −2, there was increased risk of hospital LOS ≥ 7 days (aOR: 2.08, 95% CI: 1.12, 3.89; *p* = 0.021), mechanical ventilation ≥48 h (aOR: 2.63, 95% CI: 1.32, 5.24; *p* = 0.006) and of requiring ≥3 inotropes post-operatively (aOR: 3.00, 95% CI: 1.37, 6.59; *p* = 0.006).

**Conclusion:** In children undergoing congenital heart surgery, WAZ ≤ −2 is associated with higher 30-day mortality, while HAZ ≤ −2 is associated with longer durations of hospital LOS and mechanical ventilation, and increased risk of use of 3 or more inotropes post-operatively. Future studies are necessary to develop safe and efficacious peri-operative nutritional interventions, particularly in patients with WAZ and HAZ ≤ −2.

## Introduction

Malnutrition in children has been shown to negatively impact growth and neurocognitive development (1). This is particularly evident in children with congenital heart disease (CHD) (2). Malnutrition is common in children with CHD due to increased energy requirements in addition to poor feeding and inadequate caloric intake (3–5). Insufficient protein and energy intake results in loss of both skeletal and myocardial muscle, contributing to cardiac decompensation, and failure (6). With progression of CHD, poor growth is exacerbated due to decreased peripheral perfusion with tissue hypoxia and acidosis, and sequelae such as recurrent pulmonary infections, and pulmonary hypertension (3, 7, 8).

Corrective cardiac surgery in patients with CHD has been demonstrated to have a positive impact on weight gain (9–12). Yet, a suboptimal nutritional state in itself may undermine the outcomes of corrective surgery, leading to increased peri-operative morbidities, and mortality (13–21). Although there are an increasing number of studies investigating the impact of nutritional status on outcomes after cardiac surgery in children, reported associations with clinical outcomes are varied (13–21). Heterogeneity in the results across various studies may be contributed by differences in the age range, cardiac lesions, co-morbidities, or genetic syndromes within their cohorts. Furthermore, anthropometric and biochemical parameters used to reflect nutritional status differ among these studies.

As nutritional status is a potentially modifiable risk factor, optimizing a child's pre-operative nutritional status could potentially lead to improved short- and long-term outcomes. We hypothesized that a malnourished pre-operative nutritional state is associated with adverse outcomes after cardiac surgery. If this hypothesis holds true, further studies can be performed to identify thresholds for nutritional optimization to improve outcomes after CHD surgery. Interventions can then be introduced to educate parents and healthcare professionals to optimize nutrition of these children before surgery in order to achieve improved outcomes. We also sought to assess which anthropometric parameter, namely, weight-for-age z-score (WAZ), height-for-age z-score (HAZ), or body mass index (BMI)-for-age z-score (BAZ), has the strongest association with post-operative outcomes. We elected to use BAZ as BMI is commonly used to reflect nutritional status in the adult population and BMI has been reported to be associated with outcomes after cardiac surgery (22–25).

## Materials and Methods

### Study Design

We performed a retrospective study of all children under 10 years old who underwent CHD surgery at the largest, tertiary, university-affiliated hospital in Singapore from 2012 to 2016. Study participants were restricted to being under 10 years of age in view of the lack of WAZ scores for children aged >10 years from the WHO Reference 2007 (26). Patients who had patent ductus arteriosus ligation only, genetic syndromes, or global developmental delay were excluded. The presence of global developmental delay may reflect an unrecognized genetic disorder, which may contribute to poor growth. The inclusion of patients with genetic syndromes into the study cohort might render study results less generalizable to non-syndromic patients with CHD. In addition, all infants with a corrected gestational age of <36 weeks at the time of surgery were excluded. Patients with multiple surgeries across the study period had each surgical encounter included separately if each encounter was a separate hospital admission. Approval for this study was obtained from the local research ethics committee and waiver of consent was granted.

### Data Collection

We retrieved data from inpatient and outpatient medical records, which included demographics, cardiac diagnosis, presence of a cyanotic lesion, type of surgery, pre-existing co-morbidities, and prematurity. Co-morbidities were identified based on the definitions for complex and non-complex chronic conditions as defined by the Virtual Pediatric Intensive Care Unit Performance System database (27). We used the Risk Adjustment for Congenital Heart Surgery (RACHS-1) score to classify surgical complexity (28).

At our institution, weight and height (≥2 years) or length (<2 years) measurements are obtained pre-operatively for all patients as per unit protocol. A digital weighing scale with height rod is used for children ≥2 years, while an infant weighing scale, and length mat are used for children <2 years. Measurements of pre-operative nutritional status included WAZ, HAZ, and BAZ which were derived using the World Health Organization (WHO) Anthro and AnthroPlus software from weight and height measurements taken just prior to surgery (26). We defined a WAZ, HAZ, or BAZ score ≤−2 as malnutrition and >−2 as normal.

### Statistical Analysis

Continuous and categorical variables were expressed as medians with interquartile ranges and frequencies with percentages, respectively.

The primary outcome variable was 30-day mortality. Secondary outcomes included intensive care unit (ICU) length of stay (LOS), hospital LOS, duration of mechanical ventilation (MV), and the number of inotropes used post-operatively. The primary predictor variables were WAZ, HAZ, or BAZ just prior to surgery. Patient characteristics and outcomes were summarized and compared statistically between weight-for-age z-score groups WAZ ≤−2 vs. WAZ > −2, height-for-age z-score groups HAZ ≤ −2 vs. HAZ > −2, and BMI-for-age z-score groups BAZ ≤ −2 vs. BAZ > −2 using Kruskal-Wallis and Chi square tests for continuous and categorical data, respectively.

Univariate logistic regression was performed to explore the association between the primary predictor variables (WAZ, HAZ, and BAZ) and clinical outcomes as categorical variables, where the outcomes were dichotomized into ICU LOS < 3 days or ≥3 days, hospital LOS < 7 or ≥ 7 days, duration of MV < 48 or ≥ 48 h and < 3 or ≥ 3 inotropes used post-operatively. For each clinical outcome, stepwise multivariable logistic regression analysis was performed (significance levels of 0.25 to enter and stay) to identify parsimonious subsets of independent predictors. Odds ratios and 95% confidence intervals were calculated adjusted for the other variables selected in the model which included age, the presence of a cyanotic cardiac lesion, the presence of any co-morbidities, and RACHS-1 score. All analyses were performed using SPSS v23 (Chicago, IL, USA) and SAS v9.4 (Cary, NC, USA).

## Results

### Study Population

**Figure 1:**
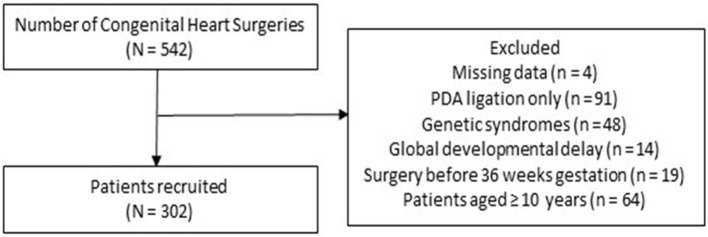
Enrollment flowchart. PDA, Patent ductus asteriosus.

Out of 542 children who underwent CHD surgery during the study period, 302 patients fulfilled inclusion criteria (Figure 1), with a median age of 16.2 [interquartile range (IQR) 3.1, 51.4)] months. Of the 302 patients, 37 (12.2%) were neonates (≤30 days), 95 (31.5%) were infants (1–12 months), and 170 (56.3%) were children (>1 year). One hundred forty-two (47%) patients were male, 37 (12.3%) were born premature, and 102 (33.8%) had cyanotic cardiac lesions. The most common cardiac lesions were ventricular septal defect (*n* = 84, 27.8%), atrial septal defect (*n* = 54, 17.9%), and Tetralogy of Fallot (*n* = 50, 16.6%) (Table 1).

**Table 1:** Cardiac lesions in our cohort (*N* = 302).

**Cardiac lesion**	***n* (%)**
Ventricular septal defect	84 (27.8)
Atrial septal defect	54 (17.9)
Tetralogy of fallot	50 (16.5)
Pulmonary atresia with ventricular septal defect	18 (6.0)
Total anomalous pulmonary venous drainage	17 (5.6)
Hypoplastic right ventricle	15 (4.9)
Double outlet right ventricle	12 (4.0)
Transposition of great arteries	9 (3.0)
Coarctation of aorta	9 (3.0)
Pulmonary atresia with intact ventricular septum	7 (2.3)
Hypoplastic left ventricle	5 (1.7)
Atrioventricular septal defect	3 (1.0)
Partial anomalous pulmonary venous drainage	3 (1.0)
Anomalous left coronary artery from the pulmonary artery	3 (1.0)
Double aortic arch	2 (0.7)
Interrupted aortic arch	2 (0.7)
Aortic stenosis	2 (0.7)
Pulmonary stenosis	2 (0.7)
Double outlet left ventricle	1 (0.3)
Aortopulmonary window	1 (0.3)
Ebstein's anomaly	1 (0.3)
Anomalous pulmonary artery from aorta	1 (0.3)
Left pulmonary artery sling	1 (0.3)

Twenty patients underwent two cardiac surgeries across the study period and each surgery was treated as a separate episode as the patients were discharged and re-admitted at a later date for the subsequent surgery.

### Nutritional Status

The median weight, height, and BMI for the entire cohort was 8.8 (IQR 4.4, 14.1) kg, 0.77 (IQR 0.57, 1.01) m, and 14.2 (IQR 12.8, 15.6) kg/m^2^, respectively. The median WAZ, HAZ and BAZ for the entire cohort was −1.46 (IQR −2.29, −0.61), −0.94 (IQR −2.10, −0.10), and −1.11 (IQR −2.19, −0.30), respectively. One hundred one patients (33.4%) had WAZ ≤ −2 while 81 (26.8%) had HAZ ≤ −2 and 88 (29.1%) had BAZ ≤ −2. Infants aged between 1 to 12 months were the most malnourished, with median WAZ −2.29 (IQR −3.11, −1.34), HAZ −1.51 (IQR −2.74, −0.45), and BAZ −1.98 (IQR −3.01, −0.96). Neonates and children were less malnourished, with median WAZ −1.26 (IQR −2.15, −0.75) vs. −0.97 (IQR −1.86, −0.21), HAZ −1.15 (IQR −2.45, −0.41) vs. −0.66 (IQR −1.64, −0.26), and BAZ −1.19 (IQR −1.48, −0.38) vs. −0.74 (IQR −1.75, 0.05), respectively.

### Outcomes

Overall median ICU and hospital LOS were 2.0 (IQR 1.0, 4.0) days and 6.0 (IQR 5.0, 14.0) days, respectively. Median duration of invasive MV was 0.65 (IQR 0.16, 2.00) days. 90% (272/302) of the cohort required 2 inotropes or less post-operatively. 6.6% (20/302) of patients developed post-operative wound complications. 4.3% (13/302) of the cohort required ECMO, of which the median duration of support was 6.7 (IQR 4.5, 12.6) days. Overall, 30-day mortality was 4.3% (13/302).

Patient characteristics and post-cardiac surgical outcomes categorized by WAZ and HAZ are shown in Tables 2, 3. Patient characteristics and post-cardiac surgical outcomes categorized according to BAZ are included in the (Supplementary Table 1).

**Table 2:** Patient characteristics and Outcomes (Categorized according to weight-for-age z-score).

**Variables**	**Weight** **Z-Score ≤−2** **(*n* = 101)**	**Weight** **Z-Score > −2** **(*n* = 201)**	***p*-value**
Age at surgery (Months)	4.7 (2.1, 24.4)	26.8 (6.7, 55.0)	0.001
Male gender	51 (50.5)	91 (45.3)	0.391
Ethnicity			0.829
Chinese	50 (49.5)	107 (53.2)	
Malay	27 (26.7)	47 (23.4)	
Indian	5 (5.0)	13 (6.5)	
Others	19 (18.8)	34 (16.9)	
**Comorbidities**			
Yes	18 (17.8)	15 (7.5)	0.010
Pulmonary	7 (6.9)	10 (5.0)	0.597
Gastrointestinal	6 (5.9)	3 (1.5)	0.065
Renal	2 (2.0)	2 (1.0)	0.409
Neurological	3 (3.0)	0 (0.0)	0.037
Endocrine	0 (0.0)	2 (1.0)	0.442
Hematological	3 (3.0)	5 (2.5)	0.536
Cyanosis	36 (35.6)	66 (32.8)	0.626
RACHS-1 category			0.410
1	16 (15.8)	43 (21.4)	
2	54 (53.5)	99 (49.2)	
3	18 (17.8)	44 (21.9)	
4 – 6	13 (12.9)	15 (7.5)	
**Outcomes**			
Duration of ICU stay (Days)	3.0 (2.0, 6.0)	2.0 (1.0, 3.0)	0.001
Duration of hospital stay (Days)	8.0 (6.0, 22.0)	6.0 (4.0, 10.5)	0.001
Duration of invasive MV (Days)	0.90 (0.43, 3.21)	0.51 (0.08, 1.68)	0.001
Number of inotrope/vasopressors			0.152
0	15 (14.8)	54 (26.9)	
1	34 (33.7)	56 (27.9)	
2	41 (40.6)	72 (35.8)	
≥3	11 (10.9)	19 (9.4)	
ECMO	7 (6.9)	6 (3.0)	0.135
Duration of ECMO (Days)	6.7 (4.7, 13.7)	6.8 (2.9, 10.7)	0.568
30-day mortality	9 (8.9)	4 (2.0)	0.012

*Continuous variables summarized in medians and interquartile ranges; categorical variables summarized in numbers and percentages. ECMO, Extra-corporeal membrane oxygenation; ICU, Intensive care unit; MV, Mechanical ventilation; RACHS-1, Risk Adjustment for Congenital Heart Surgery score*.

**Table 3:** Patient characteristics and outcomes (Categorized according to height-for-age z-score).

**Variables**	**Height Z-score** **≤-2** **(*n* = 81)**	**Height Z-score** **> −2** **(*n* = 221)**	***p*-value**
Age at surgery (Months)	6.5 (2.0, 19.0)	23.7 (4.3, 55.4)	0.001
Male gender	36 (44.4)	106 (48.0)	0.587
Ethnicity			0.917
Chinese	41 (50.6)	116 (52.5)	
Malay	22 (27.2)	52 (23.5)	
Indian	5 (6.2)	13 (5.9)	
Others	13 (16.0)	40 (18.1)	
**Comorbidities**			
Yes	14 (17.3)	19 (8.6)	0.124
Pulmonary	4 (4.9)	13 (5.9)	0.503
Gastrointestinal	6 (7.4)	3 (1.4)	0.013
Renal	2 (2.5)	2 (0.9)	0.292
Neurological	2 (2.5)	1 (0.5)	0.176
Endocrine	0 (0.0)	2 (0.9)	0.535
Hematological	3 (3.7)	5 (2.3)	0.367
Cyanosis	31 (38.3)	71 (32.1)	0.317
RACHS-1 category			0.356
1	13 (16.1)	46 (20.8)	
2	47 (58.0)	106 (48.0)	
3	12 (14.8)	50 (22.6)	
4–6	9 (11.1)	19 (8.6)	
**Outcomes**			
Duration of ICU stay (Days)	3.0 (2.0, 6.5)	2.0 (1.0, 3.0)	0.001
Duration of hospital stay (Days)	8.0 (6.0, 27.0)	6.0 (4.0, 10.0)	0.001
Duration of invasive MV (Days)	1.71 (0.37, 4.36)	0.61 (0.11, 1.39)	0.001
Number of inotrope/vasopressors			0.002
0	10 (12.3)	59 (26.7)	
1	21 (25.9)	69 (31.2)	
2	35 (43.2)	78 (35.3)	
≥3	15 (18.6)	15 (6.8)	
ECMO	4 (4.9)	9 (4.1)	0.743
Duration of ECMO (Days)	10.2 (4.3, 15.5)	6.4 (4.5, 10.0)	0.537
30-day mortality	6 (7.4)	7 (3.2)	0.118

*Continuous variables summarized in medians and interquartile ranges; categorical variables summarized in numbers and percentages. ECMO, Extra-corporeal membrane oxygenation; ICU, Intensive care unit; MV, Mechanical ventilation; RACHS-1, Risk Adjustment for Congenital Heart Surgery score*.

### Regression Analysis

#### Predictor Variable: Weight-for-Age Z-Score

Univariate logistic regression analysis demonstrated that WAZ ≤ −2 compared to WAZ > −2 was associated with an increased risk of 30-day mortality, longer duration of ICU LOS, hospital LOS, and MV.

Multivariable logistic regression analysis demonstrated that WAZ ≤ −2 compared to WAZ > −2 was associated with an increased risk of 30-day mortality (aOR: 4.01, 95% CI: 1.22, 13.13; *p* = 0.022) (Table 4).

**Table 4:** Univariate and multivariable analysis for WAZ as a predictor variable.

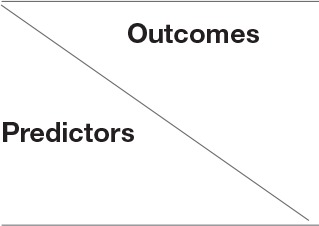	**30-day mortality**	**ICU LOS ≥3 days**	**Hospital LOS ≥7 days**	**≥3 inotropes used**	**Ventilation ≥48 hours**
			**Odds ratio** **(95% CI)** ***p*-value**		
**UNIVARIATE ANALYSIS**					
WAZ (≤ −2 vs. >−2)	4.51 (1.42, 14.3)	2.12 (1.25, 3.58)	1.81 (1.12, 2.93)	1.19 (0.55, 2.58)	1.95 (1.14, 3.35)
	**0.01**	**0.005**	**0.016**	0.661	**0.015**
Age	0.98 (0.95, 1.00)	0.96 (0.95, 0.98)	0.98 (0.97, 0.99)	0.99 (0.97, 1.00)	0.96 (0.95, 0.98)
	0.073	**<0.001**	**<0.001**	0.089	**<0.001**
Cyanosis	6.41 (1.86, 22.1)	4.61 (2.69, 7.93)	6.85 (4.02, 11.68)	5.32 (2.37, 11.9)	5.64 (3.20, 9.92)
	**0.003**	**<0.001**	**<0.001**	**<0.001**	**<0.001**
Any comorbidities	1.78 (0.42, 7.49)	1.71 (0.80, 3.65)	1.94 (0.93, 4.03)	1.40 (0.47, 4.14)	1.70 (0.78, 3.68)
	0.429	0.166	0.076	0.542	0.181
RACHS-1	**<0.001**	**<0.001**	**<0.001**	**0.001**	**<0.001**
2 vs. 1	1.96 (0.09, 42.5)	13.0 (2.44, 69.4)	8.43 (3.03, 23.4)	3.15 (0.55, 18.0)	9.63 (1.79, 51.8)
	0.667	**0.003**	**<0.001**	0.197	**0.008**
3 vs. 1	7.00 (0.35, 142)	20.2 (3.63, 113)	20.7 (6.92, 62.1)	7.80 (1.33, 45.6)	21.7 (3.90, 120)
	0.205	**0.001**	**<0.001**	**0.023**	**0.001**
4–6 vs. 1	49.4 (2.65, 917)	94.1 (15.2, 584)	67.2 (16.4, 275)	16.2 (2.61, 100)	94.1 (15.2, 584)
	**0.009**	**<0.001**	**<0.001**	**0.003**	**<0.001**
**MULTIVARIABLE ANALYSIS**^**a**^					
WAZ (≤ −2 vs. >−2)	4.01 (1.22, 13.1)				
	**0.022**				
Age		0.97 (0.95, 0.98)	0.98 (0.97, 0.99)		0.96 (0.95, 0.98)
		**<0.001**	**0.003**		**<0.001**
Cyanosis		3.27 (1.68, 6.36)	3.89 (2.11, 7.20)	5.32 (2.37, 11.9)	3.94 (1.95, 7.95)
		**0.001**	**<0.001**	**<0.001**	**0.001**
RACHS-1	**<0.001**	**0.022**	**<0.001**		**0.009**
2 vs. 1	1.74 (0.08, 36.1)	5.04 (0.90, 28.2)	4.10 (1.41, 11.9)		3.18 (0.56, 18.1)
	0.721	0.065	**0.009**		0.193
3 vs. 1	6.93 (0.35, 136)	6.67 (1.08, 41.1)	7.85 (2.40, 25.7)		6.24 (1.00, 39.0)
	0.202	**0.041**	**0.001**		**0.05**
4–6 vs. 1	41.5 (2.29, 749)	16.8 (2.40, 117)	16.7 (3.67, 76.3)		13.5 (1.89, 95.6)
	**0.012**	**0.005**	**0.001**		**0.009**

a *A stepwise algorithm was used to select variables in the multivariable model, with significance levels of 0.25 to enter and stay, based on omnibus p-values in the case of factors with multiple degrees of freedom. Empty cells in the multivariable analysis portion of the table indicate variables not selected by the stepwise procedure. ICU, Intensive care unit; LOS, Length of stay; RACHS-1, Risk Adjustment for Congenital Heart Surgery score; WAZ, Weight-for-age z-score*.

*The bold values are p-values which are statistically significant*.

#### Predictor Variable: Height-for-Age Z-Score

Univariate logistic regression analysis demonstrated that HAZ ≤ −2 compared to HAZ > −2 was associated with a longer duration of ICU LOS, hospital LOS, MV, and an increased risk of requiring ≥3 inotropes post-operatively.

Multivariable logistic regression analysis demonstrated that HAZ ≤ −2 compared to HAZ > −2 was associated with increased risk of hospital LOS ≥ 7 days (aOR: 2.08, 95% CI: 1.12, 3.89; *p* = 0.021), mechanical ventilation ≥48 h (aOR: 2.63, 95% CI: 1.32, 5.24; *p* = 0.006) and of requiring ≥3 inotropes post-operatively (aOR: 3.00, 95% CI: 1.37, 6.59; *p* = 0.006) (Table 5).

**Table 5:** Univariate and multivariable analysis for HAZ as a predictor variable.

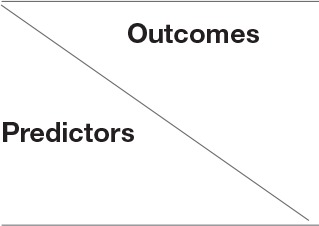	**30-day mortality**	**ICU LOS ≥3 days**	**Hospital LOS ≥7 days**	**≥3 inotropes used**	**Ventilation ≥48 hours**
			**Odds ratio** **(95% CI)** ***p*-value**		
**UNIVARIATE ANALYSIS**					
HAZ (≤ −2 vs. >−2)	2.46 (0.83, 7.30)	2.35 (1.36, 4.06)	2.17 (1.30, 3.64)	3.10 (1.45, 6.64)	2.85 (1.63, 4.99)
	0.104	**0.002**	**0.003**	**0.004**	**0.001**
Age	0.98 (0.95, 1.00)	0.96 (0.95, 0.98)	0.98 (0.97, 0.99)	0.99 (0.97, 1.00)	0.96 (0.95, 0.98)
	0.073	**<0.001**	**<0.001**	0.089	**<0.001**
Cyanosis	6.41 (1.86, 22.1)	4.61 (2.69, 7.93)	6.85 (4.02, 11.7)	5.32 (2.37, 11.9)	5.64 (3.20, 9.92)
	**0.003**	**<0.001**	**<0.001**	**<0.001**	**<0.001**
Any comorbidities	1.78 (0.42, 7.49)	1.71 (0.80, 3.65)	1.94 (0.93, 4.03)	1.40 (0.47, 4.14)	1.70 (0.78, 3.68)
	0.429	0.166	0.076	0.542	0.181
RACHS-1	**<0.001**	**<0.001**	**<0.001**	**0.002**	**<0.001**
2 vs. 1	1.96 (0.09, 42.5)	13.0 (2.44, 69.4)	8.43 (3.03, 23.4)	3.15 (0.55, 18.0)	9.63 (1.79, 51.8)
	0.667	**0.003**	**<0.001**	0.197	**0.008**
3 vs. 1	7.00 (0.35, 142)	20.2 (3.63, 113)	20.7 (6.92, 62.1)	7.80 (1.33, 45.6)	21.7 (3.90, 120)
	0.205	**0.001**	**<0.001**	**0.023**	**0.001**
4–6 vs. 1	49.4 (2.65, 917)	94.1 (15.2, 584)	67.2 (16.4, 275)	16.2 (2.61, 100)	94.1 (15.2, 584)
	**0.009**	**<0.001**	**<0.001**	**0.003**	**<0.001**
**MULTIVARIABLE ANALYSIS**^**a**^					
HAZ (≤ −2 vs. >−2)			2.08 (1.12, 3.89)	3.00 (1.37, 6.59)	2.63 (1.32, 5.24)
			**0.021**	**0.006**	**0.006**
Age		0.97 (0.95, 0.98)	0.99 (0.98, 1.00)		0.97 (0.95, 0.98)
		**<0.001**	**0.012**		**<0.001**
Cyanosis		3.27 (1.68, 6.36)	3.72 (2.00, 6.92)	5.18 (2.29, 11.7)	3.73 (1.83, 7.61)
		**0.001**	**<0.001**	**<0.001**	**0.001**
RACHS-1	**<0.001**	**0.022**	**0.001**		**0.003**
2 vs. 1	1.96 (0.09, 42.5)	5.04 (0.90, 28.2)	4.31 (1.48, 12.6)		3.51 (0.61, 20.3)
	0.667	0.065	**0.008**		0.16
3 vs. 1	7.00 (0.35, 142)	6.67 (1.08, 41.1)	8.97 (2.70, 29.8)		8.13 (1.27, 52.1)
	0.205	**0.041**	**0.001**		**0.027**
4–6 vs. 1	49.4 (2.65, 917)	16.8 (2.40, 117)	19.3 (4.16, 89.2)		17.6 (2.41, 129)
	**0.009**	**0.005**	**0.001**		**0.005**

a *A stepwise algorithm was used to select variables in the multivariable model, with significance levels of 0.25 to enter and stay, based on omnibus p-values in the case of factors with multiple degrees of freedom. Empty cells in the multivariable analysis portion of the table indicate variables not selected by the stepwise procedure. HAZ, Height-for-age z-score; ICU, Intensive care unit; LOS, Length of stay; RACHS-1, Risk Adjustment for Congenital Heart Surgery score*.

*The bold values are p-values which are statistically significant*.

#### Predictor Variable: Body Mass Index-for-Age Z-Score

Univariate and multivariable logistic regression analysis did not reveal any significant associations between BAZ and the outcomes of interest. Results are available in the (Supplementary Table 2).

## Discussion

Our study revealed a high incidence of malnutrition in patients undergoing CHD surgery (33.4% of patients with WAZ ≤ −2, 26.8% with HAZ ≤ −2 and 29.1% with BAZ ≤ −2) and a higher rate of malnutrition in infants compared to neonates or children. WAZ ≤ −2 was associated with a higher risk of 30-day mortality while HAZ ≤ −2 was associated with a higher risk of longer hospital LOS, longer duration of MV and a higher risk of requiring ≥3 inotropes post-operatively. However, BAZ did not fare as well as a predictor and was not associated with any of the outcomes of interest.

Prior to our study, several pediatric studies have examined the associations between pre-operative nutritional status and clinical outcomes (Table 6). Some have reported that low WAZ is predictive of increased post-operative mortality (13, 17, 20). A single center retrospective study from the United Kingdom (*n* = 248) found that WAZ was not associated with early mortality, but that a low WAZ predicted an increased risk of mortality at 1 year for neonates undergoing CHD surgery (13). However, the causes of late mortality were not described in the study and the reasons for a lack of association between malnutrition and early mortality remain uncertain. A large, single center, retrospective study from the United States showed that malnutrition did not impact significantly on mortality until WAZ or HAZ was ≤ -2. Further decreases in WAZ or HAZ beyond −2 were associated with proportional increases in mortality risk (20). Although our data did not demonstrate an association between low HAZ and mortality, we found that WAZ ≤ −2 was associated with an increased risk of 30-day mortality. Other studies have reported that a low WAZ and HAZ are associated with longer ICU and hospital LOS and a longer duration of MV (13, 15–17, 19, 20). Whereas, we found that a low HAZ was predictive of a prolonged hospital stay, but not a prolonged ICU LOS. This may be explained by the association between low HAZ and increased risks of both post-operative infections and longer duration of MV, which, in combination, would potentially impact more on overall LOS, rather than ICU LOS alone (20). To assess for potential contributors to ICU or hospital LOS with greater granularity in future, other complications, or outcomes that may specifically impact ICU or hospital LOS should be measured. These include hemodynamically significant cardiac arrhythmias, low cardiac output state, duration of inotrope use, post-operative infection, chylothorax, or time taken to establish enteral feeds.

**Table 6:** Summary of findings for pediatric studies employing WAZ or HAZ as predictor variables.

**References**	**Sample size (*n*)**	**Age range**	**Variable**	**Outcomes**	**Result**
Anderson et al. (15)	100	2–10 months	WAZ	- Hospital LOS - Duration of MV - Chest tube duration	- Lower WAZ predicted longer hospital LOS
Anderson et al. (16)	55	18–72 months	WAZ	- Hospital LOS - Duration of MV - Post-operative infections - Chest tube duration	- WAZ < −2 predicted increased risk of serious post-operative infections - Serious post-operative infections predicted longer hospital LOS - Duration of CPB predicted duration of MV
Wallace et al. (17)	2,747	<6 years	Age Weight WAZ	- In-hospital mortality - Fontan failure^*^ - Hospital LOS-Complications	- WAZ < −2 predicted increased in-hospital mortality, Fontan failure and longer hospital LOS - Age and weight were not significantly associated with outcome measures
Mitting et al. (13)	248	<28 days	WAZ	- Hospital mortality - Mortality at 1 year - Duration of MV/NIV - ICU LOS - Maximum lactate - Inotrope use	- Low WAZ predicted longer duration of combined MV and NIV and higher mortality at 1 year - WAZ did not impact on duration of MV
Marwali et al. (19)	249	5–36 months	WAZ	- ICU LOS - Duration of MV	- Lower WAZ was associated with longer ICU LOS and duration of MV
Ross et al. (20)	2,088	0–5 years	HAZ WAZ Weight for height Z-scores	- 30 day-Mortality - ICU LOS - Hospital LOS - Duration of MV - Infection - Cardiac arrest	- In the range of HAZ or WAZ ≤ −2, every additional unit decrease in HAZ or WAZ was associated with an 2.9% or 2.1% increased risk of mortality-Lower HAZ also predicted higher risk of cardiac arrest, infection, increased ventilation, ICU and hospital LOS - Lower WAZ predicted increased risk of cardiac arrest, infection, increased MV and ICU LOS.

*CPB, Cardiopulmonary bypass; HAZ, Height-for-age z-score; ICU, Intensive care unit; LOS, Length of stay; MV, Mechanical ventilation; NIV, Non-invasive ventilation; WAZ, Weight-for-age z-score*.

* *Fontan failure was a combination outcome, defined as either in-hospital mortality, Fontan takedown or revision*.

While WAZ provides a rough indicator of nutritional status, it lacks the ability to distinguish between body fat and muscle stores, which may have different effects on surgical outcomes (29). The limitations of measuring WAZ at a single time point should also be recognized, given that acute fluid shifts, capillary leak, and edema impact on weight measurements. It will be prudent to obtain serial measurements of body weight, while taking the clinical status of the patient into consideration, before deriving a WAZ that should be used for risk stratification. Although height is not subject to the same variability as weight, emphasis must still be placed on obtaining accurate height measurements to derive HAZ. In view of the inherent limitations of using weight or height as markers of nutrition, other pediatric studies have employed different parameters to reflect nutritional status in their comparison between malnutrition and post-operative outcomes. These parameters include triceps skin-fold thickness and other biochemical measurements. In a small prospective study involving 41 children aged 0–5 years, a lower pre-operative triceps skin-fold z-score was associated with longer duration of inotrope use, MV, and ICU LOS (18). Another small prospective study involving 30 children deemed to have high surgical risk reported that pre-operative serum albumin <3 g/dL was associated with higher mortality and post-operative infections, while post-operative serum albumin <3 g/dL was associated with longer hospital LOS. Notably, pre-operative anthropometry including HAZ, WAZ, and weight-for-height z-scores were not associated with any clinical outcomes in this study (21). Unfortunately, in our study, we were not able to investigate associations between these alternative nutritional markers and clinical outcomes. The wide range of parameters used in many of these studies reflects the challenge of assessing nutritional status at the bedside (30). There have been several attempts at developing pediatric nutritional screening tools and combined anthropometric stratification schemes (31, 32). However, these require validation in large scale studies before routine clinical use can be implemented. Future studies assessing the impact of nutrition on outcomes may need to consider including a range of nutritional parameters or nutritional screening tools which are accessible, reproducible, and cost-effective.

Ultimately, the association between nutritional parameters and clinical outcomes is complex and it is challenging to account for the differences in associations observed between WAZ, HAZ, and BAZ in our cohort. There are various categories of malnutrition. Low weight-for-height is known as wasting and usually indicates recent and severe weight loss. Low height-for-age is known as stunting, which is reflective of chronic undernutrition. Whereas, low weight-for-age is known as underweight, which may be indicative of wasting, stunting or both (33). From this, it follows that changes in weight should reflect both acute and chronic biological processes, whereas changes in height should reflect primarily chronic biological processes. This may suggest that a combination of acute and chronic physiological derangements provides a greater contribution to mortality risk in the post-operative CHD patient, accounting for the association between WAZ, and mortality in our cohort. However, we did not find WAZ to be associated with outcomes such as ICU LOS, hospital LOS and duration of MV. This may be due to the inability of WAZ to distinguish between a short child with an appropriate weight and a tall child with an inappropriately low weight, suggesting that low WAZ may be sensitive for malnutrition, but not specific for the type of malnutrition. Lung function has been reported to be directedly correlated to height and increases in height during puberty have also been associated with increases in lung capacity (34, 35). It is thus not surprising that reductions in HAZ directly reflect reduced lung capacity, accounting for the association between reduced HAZ and longer duration of MV, which would also impact on hospital LOS. It remains uncertain why BAZ was not associated with clinical outcomes in our study.

While our study findings are not novel, it reinforces the importance and utility of routine WAZ and HAZ assessment in children with CHD. This should be performed at the point of diagnosis of CHD or early before surgery, allowing for timely risk stratification and intervention by employing WAZ ≤ −2 and HAZ ≤ −2 as thresholds. Such interventions may include an individualized nutritional assessment and prescription, with the introduction of high-calorie milk formula, micronutrient, calorie or protein additives, or altering the method of feeding by employing bolus or continuous enteral nutrition via nasogastric, small bowel, or gastrostomy feeding. Therapies for gastroesophageal reflux and congestive cardiac failure must be optimized as well, to improve feed tolerance and reduce metabolic demand, respectively (14). Given the higher incidence of malnutrition amongst infants, this group of patients may benefit from more targeted pre-operative nutritional optimization, perhaps even to the extent of delaying surgery for that purpose. The opportunity for pre-operative nutritional optimization may be limited in certain cardiac lesions, where staged or definitive surgery is recommended in the neonatal period. However, neonates tend to have a poorer physiological reserve compared to infants and children and malnutrition may have a greater impact on neonates undergoing CHD surgery, especially amongst neonates that are small for gestational age (20, 36). Hence, greater emphasis must be placed on the development of safe and effective post-operative nutritional protocols for such patients, so as to mitigate post-operative physiological derangements and catabolic states (37, 38). Another “early intervention” nutritional strategy to consider is active antenatal screening for fetuses with CHD, so as to facilitate perinatal maternal nutrition optimization, in order to promote fetal weight gain and development (39, 40). This would be especially important in high risk groups, such as fetuses which are small for gestational age or those with intra-uterine growth restriction. In addition, improved maternal diet quality has also been associated with a decreased rate of certain types of CHD in children (41).

Our study has several limitations. First, the retrospective nature of our study did not allow us to employ other nutritional parameters such as body composition measures (triceps skin-fold z-score, mid-arm circumference) or serum biomarkers (pre-albumin or albumin). This limited our analysis to employing routinely collected anthropometric parameters in our clinical practice within our institution. Secondly, ours was a single institution study and it may not be possible to extrapolate our results to other populations. Thirdly, we did not collect other data that may impact post-operative outcomes, such as pre-operative functional grading of heart failure, pre-operative use of diuretics or afterload reduction, cardiopulmonary bypass time and aortic cross-clamp time or peri-operative enteral or parenteral nutritional support, which would likely confound the results of our study. Fourthly, there were very small numbers of patients with WAZ, BAZ, or HAZ > 2, limiting our ability to perform meaningful analysis to assess the outcomes of these subsets of patients. It would also have been interesting to assess the impact of nutritional status on long term outcomes such as mortality at 1 year or neurodevelopment. However, we were unable to collect such data. One of the strengths of this study is the use of the WHO references for deriving WAZ, HAZ, and BAZ. This increases the generalizability of this study as the WHO references were derived from an international cohort of children. In addition, the identification of significant co-morbidities based on the definitions for complex and non-complex chronic conditions from the Virtual Pediatric Intensive Care Unit Performance System database also increases the generalizability of our study. This reference for defining chronic conditions was selected as the various categories of chronic conditions were associated with greater odds of ICU mortality or prolonged hospital stay (27).

## Conclusion

Our study demonstrates a high prevalence of malnutrition in patients with CHD and a significant impact of malnutrition on post-operative outcomes in CHD surgery. WAZ ≤ −2 is associated with a higher risk of 30-day mortality, while HAZ ≤ −2 is associated with longer hospital LOS, longer duration of MV and higher risk of requiring 3 or more inotropes post-operatively. WAZ and HAZ must be routinely assessed in children undergoing CHD surgery, so as to facilitate identification and initiation of appropriate peri-operative nutritional interventions. Greater multi-disciplinary collaboration should be fostered to initiate perinatal maternal nutrition optimization for fetuses antenatally diagnosed with CHD, especially in high risk groups. Future prospective studies investigating which nutritional interventions have the most impact, particularly in patients with WAZ ≤ −2 and HAZ ≤ −2, are required.

## Data Availability Statement

The raw data supporting the conclusions of this manuscript will be made available by the authors, without undue reservation, to any qualified researcher.

## Ethics Statement

The studies involving human participants were reviewed and approved by SingHealth Centralized Institutional Review Board. Written informed consent from the participants' legal guardian/next of kin was not required to participate in this study in accordance with the national legislation and the institutional requirements.

## Author Contributions

CL, JL, CO, YM, TT, and JHL contributed to the writing and developing of various segments of the manuscript. RM, JA, and JW assisted with statistical analysis. JL and JHL provided mentorship to CL throughout the study period.

### Conflict of Interest

The authors declare that the research was conducted in the absence of any commercial or financial relationships that could be construed as a potential conflict of interest.
