# Supplementary material for: The Impact of Pre-operative Nutritional Status on Outcomes Following Congenital Heart Surgery
Source: Front Pediatr. 2019 Oct 23;7:429. doi: 10.3389/fped.2019.00429 (PMC6820300; doi:10.3389/fped.2019.00429)
Supplement: Supplementary file 1 [file Data_Sheet_1.docx]

***Supplementary Material***

**Supplementary Table 1.** Patient characteristics and Outcomes (Categorized according to BMI-for-age z-score)

| **Variables** | **BMI Z-Score ≤ -2**  **(n = 88)** | **BMI Z-Score > -2**  **(n= 214)** | **p-value** |
| --- | --- | --- | --- |
| Age at surgery (Months) | 6.7 (2.6, 37.7) | 19.8 (3.8, 55.3) | 0.017 |
| Male gender | 42 (47.7) | 100 (46.7) | 0.874 |
| Ethnicity |  |  | 0.506 |
| Chinese | 49 (55.7) | 108 (50.5) |  |
| Malay | 19 (21.6) | 55 (25.7) |  |
| Indian | 3 (3.4) | 15 (7.0) |  |
| Others | 17 (19.3) | 36 (16.8) |  |
| Comorbidities |  |  |  |
| Yes | 11 (12.5) | 22 (10.2) | 0.434 |
| Pulmonary | 7 (8.0) | 10 (4.7) | 0.261 |
| Gastrointestinal | 3 (3.4) | 6 (2.8) | 0.779 |
| Renal | 2 (2.3) | 2 (0.9) | 0.355 |
| Neurological | 1 (1.1) | 2 (0.9) | 0.872 |
| Endocrine | 1 (1.1) | 1 (0.5) | 0.515 |
| Hematological | 2 (2.3) | 6 (2.8) | 0.794 |
| Cyanosis | 25 (28.4) | 77 (36.0) | 0.206 |
| RACHS-1 category |  |  | 0.128 |
| 1 | 14 (15.9) | 45 (21.0) |  |
| 2 | 53 (60.2) | 100 (46.7) |  |
| 3 | 12 (13.7) | 50 (23.4) |  |
| 4 - 6 | 9 (10.2) | 19 (8.8) |  |
| **Outcomes** |  |  |  |
| Duration of ICU stay (Days) | 2.0 (1.0, 5.0) | 2.0 (1.0, 4.0) | 0.188 |
| Duration of hospital stay (Days) | 7.0 (5.0, 11.8) | 6.0 (4.0, 14.0) | 0.218 |
| Duration of invasive MV (Days) | 0.71 (0.20, 2.10) | 0.62 (0.14, 1.96) | 0.401 |
| Number of inotrope/vasopressors |  |  | 0.731 |
| 0 | 18 (20.5) | 51 (23.8) |  |
| 1 | 31 (35.2) | 59 (27.6) |  |
| 2 | 31 (35.2) | 82 (38.3) |  |
| ≥ 3 | 8 (9.1) | 22 (10.3) |  |
| ECMO | 7 (8.0) | 6 (2.8) | 0.045 |
| Duration of ECMO (Days) | 6.4 (4.7, 11.5) | 7.2 (2.9, 16.4) | 0.886 |
| 30-Day Mortality | 5 (5.7) | 8 (3.7) | 0.450 |

**Supplementary Table 1.** Continuous variables summarized in medians and interquartile ranges; categorical variables summarized in numbers and percentages. BMI: Body mass index, ECMO: Extra-corporeal membrane oxygenation, ICU: Intensive care unit, MV: Mechanical ventilation, RACHS-1: Risk Adjustment for Congenital Heart Surgery score.

**Supplementary Table 2.** Univariate and multivariable analysis for BAZ as a predictor variable

| **Outcomes**  **Predictors** | **30-Day Mortality** | **ICU LOS**  **≥ 3 Days** | **Hospital LOS ≥ 7 Days** | **≥ 3 Inotropes Used** | **Ventilation ≥ 48 hours** |
| --- | --- | --- | --- | --- | --- |
|  | **Odds Ratio**  **(95% CI)**  **p-value** | | | | |
| **Univariate analysis** | | | | | |
| BAZ (≤ -2 vs > -2) | 1.60  (0.53, 4.84)  0.405 | 1.46  (0.84, 2.52)  0.176 | 1.17  (0.71, 1.93)  0.536 | 0.90  (0.39, 2.08)  0.811 | 1.17  (0.66, 2.07)  0.592 |
| Age | 0.98  (0.95, 1.00)  0.073 | 0.96  (0.95, 0.98)  **<0.001** | 0.98  (0.97, 0.99)  **<0.001** | 0.99  (0.97, 1.00)  0.089 | 0.96  (0.95, 0.98)  **<0.001** |
| Cyanosis | 6.41  (1.86, 22.1)  **0.003** | 4.61  (2.69, 7.93)  **<0.001** | 6.85  (4.02, 11.8)  **<0.001** | 5.32  (2.37, 11.9)  **<0.001** | 5.64  (3.20, 9.92)  **<0.001** |
| Any comorbidities | 1.78  (0.42, 7.49)  0.429 | 1.71  (0.80, 3.65)  0.166 | 1.94  (0.93, 4.03)  0.076 | 1.40  (0.47, 4.14)  0.542 | 1.70  (0.78, 3.68)  0.181 |
| RACHS-1 | **<0.001** | **<0.001** | **<0.001** | **0.002** | **<0.001** |
| 2 vs 1 | 1.96  (0.09, 42.5)  0.667 | 13.0  (2.44, 69.4)  **0.003** | 8.43  (3.03, 23.4)  **<0.001** | 3.15  (0.55, 18.0)  0.197 | 9.63  (1.79, 51.8)  **0.008** |
| 3 vs 1 | 7.00  (0.35, 142)  0.205 | 20.2  (3.63, 113)  **0.001** | 20.7  (6.92, 62.1)  **<0.001** | 7.80  (1.33, 45.6)  **0.023** | 21.7  (3.90, 120)  **0.001** |
| 4 to 6 vs 1 | 49.4  (2.65, 917)  **0.009** | 94.1  (15.2, 584)  **<0.001** | 67.2  (16.4, 275)  **<0.001** | 16.2  (2.61, 100)  **0.003** | 94.1  (15.2, 584)  **<0.001** |
| **Multivariable analysis ^1^** | | | | | |
| BAZ (≤ -2 vs > -2) |  |  |  |  |  |
| Age |  | 0.97  (0.95, 0.98)  **<0.001** | 0.98  (0.97, 0.99)  **0.003** |  | 0.96  (0.95, 0.98)  **<0.001** |
| Cyanosis |  | 3.27  (1.68, 6.36)  **0.001** | 3.89  (2.11, 7.20)  **<0.001** | 5.32  (2.37, 11.9)  **<0.001** | 3.94  (1.95, 7.95)  **0.001** |
| RACHS-1 | **<0.001** | **0.022** | **0.001** |  | **0.009** |
| 2 vs 1 | 1.96  (0.09, 42.5)  0.667 | 5.04  (0.90, 28.2)  0.065 | 4.10  (1.41, 11.9)  **0.009** |  | 3.18  (0.56, 18.1)  0.193 |
| 3 vs 1 | 7.00  (0.35, 142)  0.205 | 6.67  (1.08, 41.1)  **0.041** | 7.85  (2.40, 25.7)  **0.001** |  | 6.24  (1.00, 39.0)  **0.050** |
| 4 to 6 vs 1 | 49.4  (2.65, 917)  **0.009** | 16.8  (2.40, 117)  **0.005** | 16.7  (3.67, 76.3)  **0.001** |  | 13.5  (1.89, 95.6)  **0.009** |

**Supplementary Table 2.** ^1^ A stepwise algorithm was used to select variables in the multivariable model, with significance levels of 0.25 to enter and stay, based on omnibus p-values in the case of factors with multiple degrees of freedom. Empty cells in the multivariable analysis portion of the table indicate variables not selected by the stepwise procedure. BAZ: BMI-for-age z-score, BMI: Body mass index, ICU: Intensive care unit, LOS: Length of stay, RACHS-1: Risk Adjustment for Congenital Heart Surgery score.
